# Comparative cross-sectional assessment of knowledge, attitude and practice among university students and employees towards the use of the microbiology laboratory equipment

**DOI:** 10.12688/f1000research.49923.2

**Published:** 2021-06-23

**Authors:** Muzaheed, Omar S. El-Masry, Ali A. Rabaan, Faisal Alzahrani, Amer Alomar, Faraz Ahmed Farooqi

**Affiliations:** 1 Department of Clinical Laboratory Science, College of Applied Medical Sciences, Imam Abdulrahman Bin Faisal University, Dammam, Saudi Arabia; 2 Molecular Diagnostic Laboratory, Johns Hopkins ARAMCO Healthcare, Dhahran, Saudi Arabia; 3 College of Dentistry, Imam Abdulrahman Bin Faisal University, Dammam, Saudi Arabia

**Keywords:** Knowledge, Attitude, Practice, Assessment, Survey, Microbiology, Laboratory

## Abstract

**Background: **Continuous evaluation of students and employee’s knowledge and attitude in clinical laboratories is mandatory to ensure a high level of competency, proper practice and to assess the need for training, which shall be reflected on the quality of laboratory results. The aim of the present study was to evaluate the knowledge, attitude, and practice in microbiology laboratories among employees (at King Fahd Hospital of the University) and clinical laboratory students (at Imam Abdulrahman Bin Faisal University)

**Methods:** This was a cross-sectional survey of 30 2 ^nd^ year students, 26 3 ^rd^ year students, 24 4 ^th^ year students in the Clinical Laboratory Sciences department, and 30 employees. Participants completed a survey comprising 30 questions to assess their knowledge and attitude towards the use of equipment and practice in the microbiology laboratory.

**Results:** The results indicated that there was no significant difference between the average scores of all levels of students regarding their knowledge (p = 0.85, 0.999, and 0.869), attitude (p = 0.883, 0.996, 0.853), and practice (p=0.633, 0.325, 0.858) in the microbiology laboratory. Employees scores (knowledge;5.03±2.646, attitude; 12.03±4.89, and practice; 7.7±6.11) were quite poor, as indicated by the lower average results than that of students (knowledge; 5.65±3.08, attitude; 13.25±5.33, and practice; 13.46±5.7).

**Conclusions:** It is concluded that the knowledge, attitude, and practice of students and employees in the microbiology laboratory needs to be meticulously monitored and improved to ensure high achievement of learning outcomes and better overall performance in the laboratory. This may be achieved through using frequent quizzes and continuous education programs.

## Introduction

Microbiology laboratories are unique work environments that exert potential health hazards, which requires focused knowledge and training for all users. The risk of exposure to pathogens and misuse of laboratory equipment are two important hazards related to microbiology laboratories. Throughout history, and due to the lack of safe practice and personal errors many laboratory workers have contracted infections ^1^. Similarly, mishandling of laboratory equipment, especially those operating at high temperatures or speeds can pose considerable risks to the laboratory personnel ^2^. In order to reduce these risks related to the use of equipment and practice in the microbiology laboratory, dedicated safety training and education should be in place for every laboratory ^2^.

Microbiology laboratory equipment can be broadly classified as disposable or reusable. The most common reusable components include microscopes, autoclaves, colony counters, vortex mixers, hot air ovens, refrigerators, distilled water plants, Bunsen burners, and pipettes. Laboratory students and employees should follow the related standard operating procedures (SOPs) while using this equipment, and failure to do so can cause damage to the equipment and expose laboratory personnel to a possible hazard ^3^. Likewise, the use of disposable equipment, including petri plates, pipetting tips and personal protective equipment (PPE) should be carried out in a proper manner and with care so that they do not become a cause of infection spread. This requires prior knowledge and training on how to use and dispose of these laboratory tools ^3^.

The importance of practices and attitudes of personnel working in microbiology laboratories has been highlighted in various studies ^4^. In this respect, workers should be professionally trained and participate in continuous training courses to ensure their correct and safe usage of laboratory equipment ^5^. From our point of view, the accuracy of diagnostic test results and their reliability may depend on the laboratory technician’s knowledge, attitude and standardized practice in the laboratory. The aim of the present study was to evaluate the knowledge, attitude and practice (KAP) of the students (department of clinical laboratory sciences, Imam Abdulrahman Bin Faisal University) and staff (King Fahd Hospital of the University) towards the microbiology laboratory and the use of its equipment.

## Methods

### Study design

This was a cross-sectional questionnaire-based study, conducted among the undergraduate students of the Clinical Laboratory Science Department at Imam Abdulrahman Bin Faisal university and the technical staff working in the teaching hospital of the university in Dammam, eastern province of Saudi Arabia. 

### Study instrument

A structured self-administered questionnaire was designed by the authors and reviewed by subject experts (Dr. S. Acharya., Assistant professor of microbiology and Dr. Elfadil A, Assistant professor of microbiology and Immunology) for its content, relevance, readability, and comprehension. The questionnaire was distributed to 10 randomly selected participants as preliminary pilot testing of the target population. Minor modifications were recommended by the pilot study group and were done before dissemination of the survey to the sample population. The overall Cronbach alpha value was 0.79 suggesting acceptable consistency of the questionnaire. Those who participated in the pilot study were excluded from the final analysis.

The survey was written in English and consisted of 30 questions divided into four main sections ^6^. The questionnaire started with a section asking for participants’ year of study, specialization, department, and college. Sections two to-four comprised 10 questions each to evaluate knowledge, attitudes and practice in the microbiology laboratory, respectively. All questions evaluating knowledge, attitude and practice were associated with categorical responses: yes/no/not sure.

### Participants

All students in years 2, 3 and 4 who belonged to the department of clinical laboratory sciences at Imam Abdulrahman Bin Faisal University, were asked to participate in the study. Students from other departments as well as those who had recently changed their specialty to clinical laboratory sciences were excluded from participation. All the staff of the microbiology laboratory at King Fahd Hospital of the University were asked to participate. Staff members who recently joined were excluded; only staff members with at least five years’ experience were included in the study. 

### Study procedure

The survey was hosted on the online resource “ Question Pro”. The college registrar provided the of email addresses of all pupils in each university year. The link for the survey was then sent to the students and all responses were collected between 19 ^th^ February – 03 ^rd^ March. The whole study was completed in the period completed between February 2020 – September 2020. The objectives of the research were explained to the students on the very first page and students were proceeded to take the survey only after checking the consent box. Participation was voluntary, and no benefits or incentives were given to participants. There were no personal data collected during this study.

### Statistical analysis

Data was initially exported to MS Excel 365 from the “QuestionPro” database. After cleaning the dataset was imported to Statistical Package for Social Science ( SPSS version 22, Inc. USA) for further analysis. Data was presented as frequencies, percentage, mean and SD. The knowledge sections of the questionnaire contained 10 questions and response were collected as wrong answer (marked 0) and correct answer (marked 1). Attitude and practice scores were coded as yes, don’t know and no (3, 2, 1, respectively). Overall and for each section the items internal consistency was evaluated through Cronbach's Alpha. Comparison between student’s scores was conducted using one-way analysis of variance (ANOVA), while the student independent sample t-test was employed to compare between students’ and employees’ scores. The p-value of ≤0.05 was considered statistically significant.

## Results

The questionnaire was disseminated to a target sample comprising 30 students in each study level (2 ^nd^, 3 ^rd^ and 4 ^th^ year students) and 30 employees making the total number of target participants 120; among them 110 responded ^7^. Out of 110, 30 (100%) were 2 ^nd^ year students, 26 (86.66%) were 3 ^rd^ year students, 24 (80%) were 4 ^th^ year students and 30 (100%) were employees (microbiology laboratory personnel). All of participants belonged to the same university and its teaching hospital. The response rate was 73%, incomplete or denied participation were excluded. Overall, it was found that students’ percentages of correct answer were higher than the employees. In brief the average student scores (mean±S.D) in the knowledge domain were 5.80±2.49 (2 ^nd^ year), 5.35±3.58, (3 ^rd^ year), and 5.79±3.27 (4 ^th^ year) out of a maximum of 10 points. The average scores in the attitude domain were 13.53±6.404 (2 ^nd^ year), 12.85±5.409 (3 ^rd^ year), and 13.67±3.691 (4 ^th^ year) out of a maximum of 30 points. Regarding practice, student scores were 14.60±5.73 (2 ^nd^ year), 13.16±6.34 (3 ^rd^ year) and 12.33±5.001 (4 ^th^ year) out of a maximum of 30 points. Whilst mean (SD) employees scores were 5.03±2.646 out of 10 for knowledge, 12.03±4.89 out of 30 for attitude, and 7.7±6.11 out of 30 for practice. Among the all knowledge related question the only one item (The UV-visible spectrophotometer uses light visible range) was answered correctly by the majority of the employees ( Table 1), likewise the good practice response was also higher among the students whereas the “don’t know” response for several items was higher among the employees ( Table 2). However, the attitude response of both groups was somehow similar almost for all items ( Table 3). 

**Table 1.** Participant’ knowledge about microbiology laboratory equipment.

Questions	Options	2 ^nd^-year Total=30 (%)	3 ^rd^-year Total=26 (%)	4 ^th^-year Total=24 (%)	Employers Total=30 (%)	Total (%)
Is it recommended to calibrate the pH meters before using? **Correct answer:** **Yes**	Yes	30 [100%]	23[92.31]	24[100]	30[100]	108[98%]
	No	0 [0]	[0]	[0]	[0]	0 [0%]
	Don’t know	0 [0]	3[7.69]	[0]	[0]	2[2%]
Sterilization of media and lab equipment is carried out in autoclave at 121°C? **Correct** **answer:** **Yes**	Yes	24[81.25]	12[46.15]	14[60.00]	11[36.66]	62[56%]
	No	6[18.75]	5[19.23]	0[0]	19[62.50]	29[25%]
	Don’t know	0[0]	9[34.62]	10[40.00]	0[0]	19[19%]
Centrifugation of bacterial culture is carried out to collect the bacterial cells in the supernatant. **Correct answer: No**	Yes	8[25]	11[42.31]	10[40.00]	22[75.00]	51[46%]
	No	6[18.75]	8[30.77]	10[40.00]	8[25.00]	31[29%]
	Don’t know	16[56.25]	7[26.92]	4[20.00]	0[0]	29[26%]
Can a vortex be used to separate particles based on their sizes? **Correct answer: No**	Yes	11[37.50]	11[42.31]	5[20.00]	26[87.50]	53[47%]
	No	4[12.50]	10[38.46]	14[60.00]	4[12.50]	32[31%]
	Don’t know	15[50.00]	5[19.23]	5[20.00]	0[0]	25[22%]
Polymerase chain reaction (PCR) is a DNA amplification technique? **Correct answer: ** **Yes**	Yes	30[100]	22[84.62]	24[100]	30[100]	106[96%]
	No	0[0]	3[11.54]	0[0]	0[0]	3[3%]
	Don’t know	0[0]	1[3.85]	0[0]	0[0]	1[1%]
While carrying out a microscopical examination, is it recommended to change the lens power in an ascending order, i.e. 4X, 10X, 40X? **Correct answer: Yes**	Yes	30[100]	19[73.08]	19[80.00]	24[80]	92[83%]
	No	0[0]	3[11.54]	5[20.00]	0[0]	8[8%]
	Don’t know	0[0]	4[15.38]	0[0]	6[20]	10[9%]
Would we use an electronic cell counter to determine the number of thrombocytes in a ul of blood? **Correct answer: Yes**	Yes	9[31.25]	9[34.62]	10[40.00]	8[25.00]	35[33%]
	No	8[25]	4[15.38]	5[20.00]	19[62.50]	36[31%]
	Don’t know	13[43.75	13[50.00]	9[40.00]	3[12.50]	39[37%]
The UV-visible spectrophotometer uses light visible range between 400 – 700 nm of electromagnetic radiation spectrum. **Correct ** **answer: Yes**	Yes	13[43.75]	18[69.23]	10[40.00]	24[80]	65[58%]
	No	6[18.75]	4[15.38]	0[0]	6 [20]	16[14%]
	Don’t know	11[37.50]	4[15.38]	14[60.00]	0[0]	30[28%]
A temperature precision vortex mixture is the best equipment for carrying out immunochemical reactions that require light mixing. **Correct answer: Yes**	Yes	17[56.25]	6[23.08]	4[20.00]	8[25.00]	35[31%]
	No	6[18.75]	3[11.54]	10[40.00]	0[0]	18[18%]
	Don’t know	7[25.00]	17[65.38]	10[40.00]	22[75.00]	57[51%]
Are circulating-water baths ideal for experiments which require temperature precision? **Correct answer: Yes**	Yes	11[37.50]	12[46.15]	10[40.00]	4[12.50]	37[34%]
	No	2[6.25]	5[19.23]	4[20.00]	0[0]	12[11%]
	Don’t know	17[56.25]	9[34.62]	10[40.00]	26[87.50]	62[55%]

**Table 2.** Participant’ practice while using microbiology laboratory equipment.

Questions	Options	2 ^nd^-year Total=30 (%)	3 ^rd^-year Total=26 (%)	4 ^th^-year Total=24 (%)	Employers Total=30 (%)	Total (%)
Can distilled water or deionized water be used for storing pH electrode? **Correct** **answer: No**	Yes	19[62.50]	13[50.00]	14[60.00]	26[87.50]	72[65%]
	No	11[37.50]	5[19.23]	0[0]	0[0]	16[14%]
	Don’t know	0[0]	8[30.77]	10[40.00]	4[12.50]	21[21%]
For analyzing a chemical reaction, the change in color can be observed by using a spectrophotometer. **Correct answer: Yes**	Yes	24[81.25]	25[96.15]	24[100]	19[63.33]	92[85%]
	No	4[12.50]	0[0]	0[0]	0[0]	4[3%]
	Don’t know	2[6.25]	1[3.85]	0[0]	11[36.6]	14[12%]
Is it safe to work in a biological safety cabinet (BSC) when the UV light is on? **Correct answer: No**	Yes	6[18.75]	7[26.92]	0[0]	18[62.50]	31[27%]
	No	15[50.00]	11[42.31]	10[40.00]	4[12.50]	39[36%]
	Don’t know	9[31.25]	8[30.77]	14[60.00]	8[25.00]	39[37%]
Do you sterilize your inoculating loop before as well as after using it? **Correct** ** answer: Yes**	Yes	28[93.75]	25[96.15]	24[100]	12[40]	89[82%]
	No	0[0]	0[0]	0[0]	8[26.66]	8[7]
	Don’t know	2[6.25]	1[3.85]	0[0]	10 [33.33]	13[11%]
Do you set the denaturation temperature in PCR in accordance with the melting temperatures of the primers that are being used? **Correct answer: Yes**	Yes	26[87.50]	15[57.69]	10[40.00]	4[12.50]	55[49%]
	No	4[12.50]	4[15.38]	4[20.00]	0[0]	13[12%]
	Don’t know	0[0]	7[26.92]	10[40.00]	26[87.50]	43[39%]
For getting the best resolution through a lab microscope, is use of oil immersion a viable option? **Correct answer: Yes**	Yes	17[56.25]	17[65.38]	19[80.00]	11[36.6]	64[60%]
	No	9[31.25]	4[15.38]	0[0]	2[6.6]	15[13%]
	Don’t know	4[12.50]	5[19.23]	5[20.00]	17[62.50]	32[29%]
If you are given a blood sample to detect whether the person is allergic to something, would you use Electronic cell counter for the quick determination of the number of neutrophils? **Correct answer:** ** Yes**	Yes	19[62.50]	12[46.15]	6[25.00]	8[25.00]	44[40%]
	No	2[6.25]	6[23.08]	6[25.00]	0[0]	14[14%]
	Don’t know	9[31.25]	8[30.77]	12[50.00]	22[75.33]	52[47%]
Will a shaking water bath be ideal equipment for growing bacteria in broth culture media? **Correct answer: Yes**	Yes	11[37.50]	4[15.38]	0[0]	4[12.50]	19[16%]
	No	13[43.75]	8[30.77]	14[60.00]	0[0]	36[34%]
	Don’t know	6[18.75]	14[53.85]	10[40.00]	26[87.50]	55[50%]
Will you use a hot plate when you have to prepare a buffer of certain pH? **Correct** **answer: Yes**	Yes	24[81.25]	18[69.23]	10[40.00]	4[12.50]	56[51%]
	No	2[6.25]	1[3.85]	4[20.00]	23[75.00]	30[26%]
	Don’t know	4[12.50]	7[26.92]	10[40.00]	3[12.50]	24[23%]
Do you monitor the temperature accuracy of lab incubators by placing thermometer in them? **Correct answer: Yes**	Yes	9[31.25]	14[53.85]	24[100]	11[36.6]	58[55%]
	No	11[37.50]	8[30.77]	0[0]	0[0]	19[17%]
	Don’t know	10[31.25]	4[15.38]	0[0]	19[63.33]	33[28%]

**Table 3.** Participants’ attitude towards microbiology laboratory equipment.

Questions	Options	2nd-year Total=30 (%)	3rd-year Total=26 (%)	4th-year Total=24 (%)	Employers Total=30 (%)	Total (%)
Do you think it is ideal to use a water bath when heating of a flammable compound is required? **Correct answer: Yes**	Yes	11[37.50]	4[15.38]	5[20.00]	8[25.00]	28[24%]
	No	13[43.75]	19[73.08]	14[60.00]	22[75.00]	69[63%]
	Don’t know	6[18.75]	3[11.54]	5[20.00]	0[0]	13[13%]
While using a hot plate, will you start the magnetic stirring before placing the magnetic stirrers in the mixture? **Correct** **answer: No**	Yes	8[25.00]	2[7.69]	0[0]	22[75.00]	32[27%]
	No	9[31.25]	6[23.08]	5[20.00]	4[12.50]	24[22%]
	Don’t know	13[43.75]	18[69.23]	19[80.00]	4[12.50]	54[51%]
Do you feel it is ok to place your food items in the laboratory fridge and freezers? **Correct answer: No**	Yes	4[12.50]	0[0]	0[0]	19[62.50]	23[19%]
	No	26[87.50]	26[100]	24[100]	11[37.50]	88[81%]
	Don’t know	0[0]	0[0]	0[0]	0[0]	0[0]
Do you think it is a necessary that laboratory ovens have a vent that is directly connected to the exhaust system? **Correct** **answer: Yes**	Yes	19[62.50]	13[50.00]	19[80.00]	8[25.50]	59[55%]
	No	4[12.50]	3[11.54]	5[20.00]	4[12.50]	15[14%]
	Don’t know	7[25.00]	10[38.46]	0[0]	18[60]	35[31%]
From safety point of view do you think hot plates are better than water baths. **Correct** **answer: No**	Yes	9[31.25]	8[30.77]	10[40.00]	18[62.50]	46[41%]
	No	9[31.25]	10[38.46]	5[20.00]	8[25.00]	32[29%]
	Don’t know	12[37.50]	8[30.77]	9[40.00]	4[12.50]	33[30%]
Do you think it is necessary to balance the load while using a centrifuge? **Correct** **answer: Yes**	Yes	26[87.50]	24[92.31]	24[100]	30[100]	104[95%]
	No	2[6.25]	1[3.85]	0[0]	0[0]	3[3%]
	Don’t know	2[6.25]	1[3.85]	0[0]	0[0]	3[3%]
While pipetting is it necessary to use a pipette bulb or proper pipetting devices for transferring liquid cultures? **Correct** **answer: Yes**	Yes	24[81.25]	22[84.62]	14[60.00]	6[18.75]	66[61%]
	No	6[18.75]	3[11.54]	5[20.00]	24[81.25]	38[33%]
	Don’t know	0[0]	1[3.85]	5[20.00]	0[0]	6[6%]
If autoclave is not available, do you think it is effective to disinfect the apparatus with 10% bleach for at least 1 to 2 hours? **Correct answer: Yes**	Yes	11[37.50]	6[23.08]	0[0]	8[25.00]	25[21%]
	No	4[12.50]	6[23.08]	5[20.00]	0[0]	15[14%]
	Don’t know	15[50.00]	14[53.85]	19[80.00]	22[75.00]	71[65%]
Do you think it is the best option to use a syringe filter for removing microbes from a small sample that cannot be sterilized through autoclaving? **Correct answer: Yes**	Yes	17[56.25]	10[38.46]	10[40.00]	8[25.00]	44[40%]
	No	8[25.00]	9[34.62]	10[40.00]	18[60]	44[40%]
	Don’t now	5[18.75]	7[26.92]	4[20.00]	4[12.50]	21[20%]
Do you think it is necessary to clean the residual pitch in the distillation plant? **Correct answer: Yes**	Yes	23[75.00]	20[76.92]	24[100]	26[87.50]	93[85%]
	No	2[6.25]	1[3.85]	[0]	0[0]	3[3%]
	Don’t know	5[18.75]	5[19.23]	[0]	4[12.50]	14[13%]

### Internal consistency

The items (questions) used in the current study to assess the performance of students and employees in knowledge, attitude and practice domains are summarized in Table 1, Table 2, Table 3. The correct answer for each question was indicated in the tables along with the listed options for the participants. The reliability of the questionnaire items used to assess knowledge, attitude and practice was evaluated by assessing the internal consistency of each domain’s questions by calculating the Cronbach's Alpha ( Table 4). The values of the Cronbach's Alpha coefficient for knowledge, attitude and practice were 0.888, 0.916, and 0.932, respectively, indicating that the items in each group are closely related as a set of questions.

**Table 4.** Cronbach's Alpha coefficient values indicating the internal consistency of questionnaire items.

Domain	Cronbach's Alpha	No. of Items
Knowledge	0.888	10
Attitude	0.916	10
Practice	0.932	10

### Descriptive data for knowledge, attitude and practice scores

The overall average scores of the three domains for the whole study sample are shown in Table 5 and the average performance scores for each group are shown in Table 6. Among the students, the 2nd year students who are the most junior students in this study had the highest level of knowledge (5.80±2.49), followed by the most senior 4th year (5.79±3.27) and 3rd year students (5.35±3.58). Faculty surprisingly had the lowest knowledge score among the participants (5.03±2.64). Likewise, the average scores of the 4th year and the 2nd year students in attitude-related questions were close (13.67±3.69 and 13.53±6.40, respectively). The lowest score in the attitude domain was reported for the 3rd year students followed by the employees (12.85±5.40 and 12.03±4.89). Regarding practice-related questions, the 2nd year students achieved the best scores (14.6±5.73), followed by the 3rd year (13.16±6.34) and the 4th year students (12.33±5.00). The employees score was the least, and surprisingly, low in this domain also (7.7±6.11).

**Table 5.** Descriptive statistics of knowledge, attitude, and practice scores for whole sample.

Domain (total responses)	N	Minimum	Maximum	Mean	Std. Deviation
Knowledge	110	0	10	5.48	2.973
Attitude	110	1	20	12.99	5.212
Practice	110	0	20	11.89	6.368
Valid N (listwise)	110				

**Table 6.** Average scores (mean±S.D) of students and employees towards knowledge, attitude and practice.

Group	Knowledge (mean±SD)	Attitude (mean±SD)	Practice (mean±SD)
2nd Year Students	5.80±2.49	13.53±6.404	14.60±5.73
3rd Year Students	5.35±3.58	12.85±5.409	13.16±6.34
4th year Students	5.79±3.27	13.67±3.691	12.33±5.001
Employees	5.03±2.646	12.03±4.89	7.7±6.11

### Comparison of student groups’ scores in knowledge-, attitude- and practice-related questions

The average scores of 2 ^nd^, 3 ^rd^, and 4 ^th^ year students in the three domains were compared using one-way analysis of variance ANOVA ( Table 7). The results indicated that there was no significant difference between the average knowledge scores of: the 2 ^nd^ and 3 ^rd^ year students (p = 0.85); the 2 ^nd ^and 4 ^th ^year students (p = 0.999); and the 3 ^rd^ and 4 ^th^ year students (p = 0.869). There was similarly a non-significant difference observed for the average attitude scores between the above-mentioned groups (p=0.883, 0.996, and 0.853, respectively), as well as for practice (p= 0.633, 0.325, and 0.858, respectively).

**Table 7.** ANOVA test results for knowledge, attitude, and practice comparisons between students.

Domain	2 ^nd^ year	3 ^rd^ year	p-value	2 ^nd^ year	4 ^th^ year	p-value	3 ^rd^ year	4 ^th^ year	p-value
Knowledge	5.80±2.49	5.35±3.58	0.85	5.80±2.49	5.79±3.27	0.999	5.35±3.58	5.79±3.27	0.869
Attitude	13.53±6.404	12.85±5.409	0.883	13.53±6.404	13.67±3.691	0.996	12.85±5.409	13.67±3.691	0.853
Practice	14.60±5.73	13.16±6.34	0.633	14.60±5.73	12.33±5.001	0.325	13.16±6.34	12.33±5.001	0.858

*p<0.05 considered statistically significant

### Comparison of student groups’ and employees’ scores in knowledge-, attitude- and practice-related questions

The independent sample t-test ( Table 8) was used to compare the KAP scores between employees and students. The results indicated a non-significant difference between students and employees regarding knowledge or attitude (p=0.335 and 0.24, respectively). On the other hand, the difference between students and employees’ scores for practice-related questions was significantly different (13.46±5.7 and 7.70±6.11, respectively, p<0.0001).

**Table 8.** Independent t-test results for knowledge, attitude, and practice comparisons between students and employees.

Domain	Students (mean±SD)	Employees (mean±SD)	p-value
Knowledge	5.65±3.08	5.03±2.54	0.335
Attitude	13.25±5.33	12.03±4.83	0.24
Practice Score	13.46±5.7	7.70±6.11	<0.0001

*p<0.05 considered statistically significant

## Discussion

The present survey was carried out to evaluate and analyze three domains: knowledge, attitude and practice towards equipment used in the microbiology lab by university students and employees. The results of the current study revealed that there was no significant difference between students in different years regarding their KAP in the microbiology lab. Of note, employees had the lowest scores amongst the studied groups. In this respect, employee’s average scores for practice-related questions were significantly different from the average score of students in the same domain. Similar findings have been reported by Jairoun and colleagues ^8^ who evaluated KAP of medical students (MS) and non-medical students (NS) towards the use of antibiotics in the United Arab Emirates (UAE). They reported that medical students had better knowledge of antibiotics and their side effects compared to the non-medical students; however, employees showed the least knowledge when compared to the students. Since employees mostly do routine work, they may not bother to get more awareness about laboratory equipment as noticed from the assessment of the employees’ KAP regarding health precautions in the laboratory. This is disappointing as they play a very important role in regulating daily tasks of the laboratory; this may compromise laboratory results and jeopardize health management outcomes in patients. In addition, employees play a significant role in students’ training.

In another context, Barikani ^9^, stated that around 50% of the students surveyed had knowledge of 'hand washing before and after using gloves", but only 40% developed the attitude to do that. Moreover, the number of students who practiced hand washing was reduced to 16.2%, which showed the non-serious behavior of the students. In our opinion, this behavior can be explained based on the fact that with seniority, students tend to ignore rules and instructions and become more careless towards the usage of equipment. Students’ attitudes were analyzed through a questionnaire and not based on observation and therefore reflect their subjective views. From the results, it can be stipulated that students disregard proper attitude protocols towards the use of laboratory. Also, in the final years of the program, especially in level four, students’ courses are more focused towards practical skills and hospital rotations, which might affect student attention to basic practice knowledge taught in the foundation years of the program.

The lab employees who participated in the current survey showed the lowest average in all three KAP domains. The results may be explained, from our own perspective, by the tendency of most employees to ignore laboratory-related instructions, and this might be reflected in their attitude and practice. Also, the low average scores of employees in the knowledge domain might be explained by the lack of continuous training and participation in educational programs. In this context, we understand that some employees might have forgotten about using different technique, and the reason behind it might be the low frequency of such procedures and/or the expansion in laboratory automation. Similar results were reported by Ejilemele and Ojulu ^2^, who carried out a KAP survey in pathology laboratory staff. They reported gross insufficiencies in the KAP of safety protocols by the laboratory staff in different microbiology areas. It was found that laboratory employees lack the required KAP about safe specimen collection, standard use of PPE, and usage of centrifuge and first aid kits.

The current study was also designed to analyze the students’ approach and practice towards facilities/equipment they are provided with. It might be concluded from the results of the current study that knowledge and attitude affect the practice. Results showed that students’ achievement in all levels was comparative, though ideally, KAP should be higher in more senior students as skills-related learning outcomes and capacity of laboratory training are introduced in much higher weights in final years of study, which would be reflected in their knowledge and practical skills. This means that higher levels students might become irresponsible and less serious towards rules and concepts with time, though the difference was not statistically significant. These findings are in agreement with the findings of behavioral survey carried out by Askarian and colleagues ^10^. They reported that the carelessness in behavior was observed in Iranian medical students towards practice of standard isolation precautions. When they were tested for precautionary measures taken, most of them did not know about the recommended disinfecting techniques. Another study also showed that knowledge and attitudes among medical students were acceptable but practices towards standard isolation precautions was poor ^11^.

It was also observed in the current study that employees’ scores were markedly lower for practice than those for students, which means they have lesser knowledge of equipment and laboratory management. The result revealed their incompetence compared with students. Probably, the lack of dedication and problematic conduct directly affect employee’s own safety and health, as well as students. These findings are alarming, as employees should supposedly have better knowledge, attitude, and practice of correct laboratory procedures than students. Students may be careless as they are learning, but it should not be tolerated by supervisors. Our findings are in harmony with a survey carried out by Zaveri and Karia ^12^ who analyzed the KAP of laboratory technicians regarding standard precautions using a cross-sectional study. They reported that health care workers (technicians directly involved with the work in the laboratories of selected hospitals) showed poor knowledge, attitude, and practices of universal work precautions that are defined, according to the center for disease control, as precautions to prevent blood borne infections to workers who provide first-aid or health services ^6^.

## Conclusion

These findings highlight the importance of this and similar surveys that help us to evaluate the status of knowledge, attitude and practice of laboratory students and employees in standard microbiology practice. Based on the results, it can be suggested that lab employees should be trained so that they are not only present to keep an eye on students, but are qualified to provide help and guidance to students regarding experiments, equipment usage, cleanliness and safety. This study also highlights the need for regular educational courses for lab employees to keep them updated about the latest equipment and any new practices. Moreover, we propose that students should be evaluated regularly on their learning and attitudes, as ensuring the right attitude and practice towards microbiology equipment is necessary for the safety of both users and equipment, especially in more senior students.

To this end, the authors may conclude that as university students progress through their degree, their knowledge and attitude may not necessarily improve; learning and good conduct cannot be proportional to passing classes. In this context, we may suggest that knowledge, attitude and practice develop by motivation and determination. The present study showed that commitment of students towards knowledge and practice is directly proportional to their attitude. From this study, we conclude that it is particularly important to evaluate the learning process of students and employees and they should be regularly assessed.

### Study limitations

The conclusion from the current study was mainly based on questionnaire data, which may not reflect evidence-based practice of both studied groups (students and employees). Therefore, combining questionnaires with laboratory observations could reflect the better picture of KAP in the clinical laboratory. Also, the current study evaluated KAP domains in a single batch of clinical laboratory science students and focused on microbiology instruments, which means that the findings cannot be generalized to different students and employee populations.

## Data availability

### Underling data

Harvard Dataverse: Comparative cross-sectional assessment of knowledge, attitude and practice among university students and employees towards the use of the microbiology laboratory equipment. https://doi.org/10.7910/DVN/4JHK2W ^7^.

This project contains the following underlying data:

- Raw data excel file

### Extended data

Harvard Dataverse: Comparative cross-sectional assessment of knowledge, attitude and practice among university students and employees towards the use of the microbiology laboratory equipment. https://doi.org/10.7910/DVN/4JHK2W ^7^.

This project contains the following extended data:

- Questionnaire

Data are available under the terms of the Creative Commons Zero "No rights reserved" data waiver (CC0 1.0 Public domain dedication).

## Ethical approval

Upon discussion with a review board representative, ethical approval for this study was not applied for since the data collected was all anonymous and did therefore not violate privacy or confidentiality of the participants.

## Consent

Participants were asked about their willingness to participate on the first-display page of the questionnaire which informed them that their participation was voluntary and presented a tick box to provide consent.
