# Supplementary material for: Comparative cross-sectional assessment of knowledge, attitude and practice among university students and employees towards the use of the microbiology laboratory equipment
Source: F1000Res. 2021 Jun 23;10:117. Originally published 2021 Feb 16. [Version 2] doi: 10.12688/f1000research.49923.2 (PMC8246305; doi:10.12688/f1000research.49923.2)
Supplement: Peer review history

# Peer review: Reviewer response for version 2

**DOI:** 10.5256/f1000research.57901.r83796

**Published:** 2021-07-16

**Authors:** Kamran Soomro

**Affiliations:** 1 Computer Science Research Centre, University of the West of England, Bristol, UK

Overall it is an interesting piece of work and the paper is well-written and presented. However, there are numerous typos strewn throughout the text that should be removed and citations need to be properly formatted. For example, on Page 5, the citation should be Barikani and Afaghi.

**Page 1:**

 It is unclear to me what you mean by knowledge and attitude. Does knowledge mean learning here? As in what students have learned so far?

 How do you define attitude?

**Page 3, Introduction:**

 I think this section can be made a lot leaner. IMHO it is unnecessarily verbose and repetitive.

 Also, I think you need to define what you mean by practice and attitude and state how you measure knowledge, attitude and practice.

**Page 4, Discussion:**

 Overall this section is a bit repetitive and could be streamlined significantly.

 You have also highlighted some important areas of concern for the university that merit further investigation into their causes. For example, why do 4th year students develop a relaxed attitude towards proper procedures? You have presented your opinion but I would argue that this could be a future direction for research. Similarly, there are other such important questions that I would like to see highlighted as possible directions for future research.

**Page 9, para 1:**

 It’s also possible that there is a cultural problem in the university and senior students adopt those poor cultural practices from their teachers. This probably needs more investigation and addressing at an institutional level.

 I would also argue that the findings of Askarian et al. are different findings altogether from yours. If the students did not know about the precautionary measures, that suggest failure on the part of the universities to sufficiently train and educate them. In this scenario, however, it is not clear whether the university shares any responsibility for the students’ lack of adherence to proper procedures.

**Page 9, column 2, para 2:**

 You claim that, "The present study showed that commitment of students towards knowledge and practice is directly proportional to their attitude." However, I do not see any evidence supporting this conclusion.

 For example, you claim that 2nd year students are more knowledgeable and adhere more to proper procedures. However, there is no statistically significant difference between the 2nd and 4th year students in terms of the three attributes. So how do you support this claim?

Is the work clearly and accurately presented and does it cite the current literature?

Yes

If applicable, is the statistical analysis and its interpretation appropriate?

Partly

Are all the source data underlying the results available to ensure full reproducibility?

Yes

Is the study design appropriate and is the work technically sound?

Partly

Are the conclusions drawn adequately supported by the results?

Partly

Are sufficient details of methods and analysis provided to allow replication by others?

Yes

Reviewer Expertise:

Machine Learning, Smart Cities

I confirm that I have read this submission and believe that I have an appropriate level of expertise to confirm that it is of an acceptable scientific standard, however I have significant reservations, as outlined above.

# Peer review: Reviewer response for version 2

**DOI:** 10.5256/f1000research.57901.r88111

**Published:** 2021-06-30

**Authors:** Pathiyil Ravi Shankar

**Affiliations:** 1 IMU Centre for Education, International Medical University, Kuala Lumpur, Malaysia

The authors have answered my queries and I can now approve this study.

Is the work clearly and accurately presented and does it cite the current literature?

Yes

If applicable, is the statistical analysis and its interpretation appropriate?

Yes

Are all the source data underlying the results available to ensure full reproducibility?

Yes

Is the study design appropriate and is the work technically sound?

Yes

Are the conclusions drawn adequately supported by the results?

Yes

Are sufficient details of methods and analysis provided to allow replication by others?

Yes

Reviewer Expertise:

Rational use of medicines Pharmacovigilance Questionnaire based research Health Professions Education

I confirm that I have read this submission and believe that I have an appropriate level of expertise to confirm that it is of an acceptable scientific standard.

# Peer review: Reviewer response for version 1

**DOI:** 10.5256/f1000research.53005.r86974

**Published:** 2021-06-15

**Authors:** Pathiyil Ravi Shankar

**Affiliations:** 1 IMU Centre for Education, International Medical University, Kuala Lumpur, Malaysia

The study is well-written, and the table is well presented.

 The period of study was nearly seven months which is long for a cross-sectional study. Participants may have communicated with each other which can influence the results.

 I am confused about whether a sample of respondents was chosen or if all respondents were invited to participate?

 Does the sample chosen represent the population of students and employees in terms of demographic characteristics?

 The quality of written English is mostly good. However, corrections may be required in a few places.

**Results**

 I wanted to confirm that the employees involved in the study were all faculty members for the clinical laboratory sciences course.

 What percentage of students from different years and employees participated in the study?

 The authors should try to explain better why the knowledge of students did not improve as they progressed through the course.

 What were the qualifications of the employees? How are they selected? Is there no refresher training provided to them? Do they have annual appraisals conducted?

 Could the level of English proficiency of the employees affected their response and the scores obtained?

Is the work clearly and accurately presented and does it cite the current literature?

Yes

If applicable, is the statistical analysis and its interpretation appropriate?

Yes

Are all the source data underlying the results available to ensure full reproducibility?

Yes

Is the study design appropriate and is the work technically sound?

Yes

Are the conclusions drawn adequately supported by the results?

Yes

Are sufficient details of methods and analysis provided to allow replication by others?

Yes

Reviewer Expertise:

Rational use of medicines Pharmacovigilance Questionnaire based research Health Professions Education

I confirm that I have read this submission and believe that I have an appropriate level of expertise to confirm that it is of an acceptable scientific standard, however I have significant reservations, as outlined above.

# Response

**Published:** 2021-06-23

**Authors:** . Muzaheed

**Affiliations:** Imam Abdulrahman Bin Faisal University, Saudi Arabia

Firstly, we thank the reviewer for reviewing our article and providing constructive and positive comments. We have revised our manuscript to address all comments without compromising the writing integrity. Please, find below our responses to reviewer comments.

**Reviewers’ comment:** The study is well-written, and the table is well presented.

**Author’s Response:** Thank you for your admiration.

**Reviewers’ comment:** The period of study was nearly seven months which is long for a cross-sectional study. Participants may have communicated with each other which can influence the results.

**Author’s Response:** Thank you for your valuable comments we are aware that cross-sectional data should be collected in a short time as much as possible to avoid any bias as mentioned; however, we would like to indicate that the seven months’ time was the time taken for the whole study including data analysis and writing up of the manuscript. We have revised our manuscript to make it clearer to the readers. Also, all participants who were included in the pilot study were removed from participation.

**Reviewers’ comment:** I am confused about whether a sample of respondents was chosen or if all respondents were invited to participate?

**Author’s Response:** We conducted a pilot study by choosing a random sample to validate our survey then all the participants were invited and those who responded were included in the study without selection except those who participate in the pilot study. We hope that this is clear and addressed this comment.

**Reviewers’ comment:** Does the sample chosen represent the population of students and employees in terms of demographic characteristics?

**Author’s Response:** We tried to make the study sample as representative as possible by inviting all students in levels 2, 3, and 4 also all employees in the microbiology laboratory who met our inclusion criteria were given the chance to participate in our study as described in the method’s section.

**Reviewers’ comment:** The quality of written English is mostly good. However, corrections may be required in a few places.

**Author’s Response:** We revised our article to correct any potential technical error.

**Reviewers’ comment:** I wanted to confirm that the employees involved in the study were all faculty members for the clinical laboratory sciences course.

**Author’s Response:** Yes, they are graduates of the clinical laboratory sciences program.

**Reviewers’ comment:** What percentage of students from different years and employees participated in the study?

**Author’s Response:** Thank you for the comment. In response to this comment, we have mentioned the total numbers of students and employees who were invited to participate as well as the numbers who responded to the survey as indicated at the beginning of the results section. We will update the article by including the percentage also.

**Reviewers’ comment:** The authors should try to explain better why the knowledge of students did not improve as they progressed through the course.

**Author’s Response:** Thank you for the valuable comment; we have tried to elaborate on this, please check.

**Reviewers’ comment:** What were the qualifications of the employees? How are they selected? Is there no refresher training provided to them? Do they have annual appraisals conducted?

**Author’s Response:** The employees are graduates of the clinical laboratory sciences program. They have been selected according to the criteria mentioned in the methods section. As far as we are aware, There are “Continuous Education Programs” as well as the annual appraisal.

**Reviewers’ comment:** Could the level of English proficiency of the employees affected their response and the scores obtained?

**Author’s Response:** All clinical laboratory sciences graduates should provide an English proficiency testing certificate before joining the program. Also teaching is in English and instructions indicated that investigators are available to answer any questions and clarify any ambiguous information. In this respect, we think that this was not a concern during the survey.

# Peer review: Reviewer response for version 1

**DOI:** 10.5256/f1000research.53005.r86979

**Published:** 2021-06-14

**Authors:** Manoj Sharma, Kavita Batra

**Affiliations:** 1 Kirk Kerkorian School of Medicine at University of Nevada, Las Vegas, Las Vegas, UT, USA; 2 Department of Environmental and Occupational Health, University of Nevada, Las Vegas, Las Vegas, NV, USA

Here is a review of the manuscript entitled “Comparative cross-sectional assessment of knowledge, attitude, and practice among university students and employees towards the use of the microbiology laboratory equipment.” The findings of this study will serve as the baseline data to evaluate learning outcomes and performance of students and staff in the microbiology laboratory settings. Despite this contribution, we believe there are opportunities to further strengthen this manuscript. We will share these in a bulleted format in the order that they appear.

**Abstract **

- Authors have included study setting (name of the institution/lab) in the purpose, which should be moved under methods.
- In methods section of the abstract: Overall sample size has not been provided; however, authors have provided the year wise breakup.
- No mention about the statistical method used has been made.
- Inclusion and exclusion criteria of the participants are missing.
- Please indicate whether the survey was web-based or paper and pencil?
- We are not sure what measure authors intend to indicate by 0.999, 0.869, 0.996, 0.853). Are these 95% CIs?
- Overall, the abstract needs revisions to be able to stand alone.

 
**Introduction**

- Introduction is concise and somewhat relevant. Authors have discussed the standard operating procedures (SOPs) and implications of not following them. However, it is very brief and includes only five references.  There is a need for more substantial literature base to support the necessity for the study.
- The knowledge, attitudes, and practice (KAP) paradigm is an older version of health behavior research and the contemporary literature has moved to theory-based paradigms.  Rationale for using the older KAP model needs to be explicated.

 
**Methods **

- Methodology particularly statistical analysis needs to be strengthened. Authors did not provide sample justification.
- In the study design subsection: when this study was conducted?
- Please replace “study instrument” with “survey instrument”
- There is a no mention of the psychometric testing of the instrument, namely validity and reliability in the methods section. Cronbach’s alphas have been provided in the results which are only indicative of internal consistency.  How the face, content, and construct validity of the instrument were assessed?  In their absence the results cannot be trustworthy.
- Was test-retest reliability done? In its absence the results cannot be trustworthy.
- There is no mention of the type of sampling technique or justification of sample size by the researchers.
- The reviewers wonder the rationale behind not using the gender question in the survey. It will be intriguing to know if there were any differences across gender.
- Researchers could have formed two groups of students (Juniors vs. Seniors) to see if there are any differences? Why first year students were not included in the study? Please provide the rationale in the eligibility criteria.
- It will be interesting to conduct a correlation test among the constructs of the survey, which is missing in the study.
- How missing data were handled has not been mentioned?
- Please spell out abbreviations when first time used in the text. For example, SD

 
**Results**

- We would encourage authors to use CHAMP guidelines (https://pubmed.ncbi.nlm.nih.gov/33514558/) for reporting.
- In the headers of all the table, please indicate the sample size.
- Tables should be limited to only show correct response percentages instead of other answers.
- Please add one more table of the correlation as suggested in the methods section above.

 
**Discussion **

- We think justification to the important results have not been provided adequately. For example, authors have discussed previous studies related to antibiotic use, which we think is out of context.   
- The discussion section is very weak and is not backed by sufficient literature review.
- Please add more on the potential value of this study.
- Several limitations remain unacknowledged, for instance generalizability, self-reporting bias etc.
- The authors write, “The conclusion from the current study was mainly based on questionnaire data, which may not reflect evidence-based practice of both studied groups (students and employees).”  The words “evidence-based” are typically used for empirically tested interventions and their use in this context is somewhat confusing.

On, the whole, in our opinion, this manuscript is not suitable for indexing in its present form.

Is the work clearly and accurately presented and does it cite the current literature?

Partly

If applicable, is the statistical analysis and its interpretation appropriate?

Partly

Are all the source data underlying the results available to ensure full reproducibility?

Partly

Is the study design appropriate and is the work technically sound?

Partly

Are the conclusions drawn adequately supported by the results?

No

Are sufficient details of methods and analysis provided to allow replication by others?

Partly

Reviewer Expertise:

Data analytics, behavioral research, infectious diseases, health promotion

We confirm that we have read this submission and believe that we have an appropriate level of expertise to state that we do not consider it to be of an acceptable scientific standard, for reasons outlined above.

## References

[1] : A CHecklist for statistical Assessment of Medical Papers (the CHAMP statement): explanation and elaboration. *Br J Sports Med* .2021; 10.1136/bjsports-2020-103652 10.1136/bjsports-2020-103652 33514558PMC9110112

# Peer review: Reviewer response for version 1

**DOI:** 10.5256/f1000research.53005.r81847

**Published:** 2021-03-30

**Authors:** Rama Bhat P

**Affiliations:** 1 Department of Biotechnology, Alva’s College, Moodbidri, Mangalore University, Moodbidri, Karnataka, India

The research article well planned and executed nicely with experimental designs. The introduction was brief and relevant to the work. Sampling method in general with multicentric experiment has accurately taken individual groups. Statistical methods followed has made the analysis more accurate. The presentation of the results is also good with tables at appropriate places. Discussion compared the results with available reports made more close to research data with level of significance. Literature were cited at respective places.

 There are few points to be noted before acceptance of the paper to be indexed:

- Corrections made were highlighted and changes required are given here. (e.g. "Petri plates" - here, Petri is the name of scientist, so it should be in capital.
- Other corrections like capital word or, " *et al."* were also marked. In the tables remove the full stop at the end of title. Then % was there in all the numbers in the table 1-3 in the last column. Remove it from second line onwards as it is shown in headline.

After the minor corrections the paper can be accepted to be indexed.

Is the work clearly and accurately presented and does it cite the current literature?

Yes

If applicable, is the statistical analysis and its interpretation appropriate?

Yes

Are all the source data underlying the results available to ensure full reproducibility?

Yes

Is the study design appropriate and is the work technically sound?

Yes

Are the conclusions drawn adequately supported by the results?

Yes

Are sufficient details of methods and analysis provided to allow replication by others?

Yes

Reviewer Expertise:

Microbiology, Immunomodulatory studies, Plant biotechnology, Biodiversity conservation. Herbal formulation, Anticancer and Antidiabetic studies with animal models, EBT

I confirm that I have read this submission and believe that I have an appropriate level of expertise to confirm that it is of an acceptable scientific standard.
